# The future of G protein‐coupled receptor therapeutics: Apelin receptor acts as a prototype for the advancement of precision drug design

**DOI:** 10.1002/ctm2.70120

**Published:** 2024-12-10

**Authors:** Wei‐Wei Wang, Su‐Yu Ji, Ping Xu, Yan Zhang, Yan Zhang

**Affiliations:** ^1^ Department of Pathology of Sir Run Run Shaw Hospital, and Department of Pharmacology,and Liangzhu Laboratory Zhejiang University School of Medicine Hangzhou China; ^2^ MOE Frontier Science Center for Brain Research and Brain‐Machine Integration Zhejiang University School of Medicine Hangzhou China; ^3^ Institute of Cytology and Genetics, School of Basic Medical Sciences Hengyang Medical School, University of South China Hunan China; ^4^ Department of Cardiology and Institute of Vascular Medicine, Peking University Third Hospital，Institute of Cardiovascular Sciences, School of Basic Medical Sciences, Peking University Health Science Center，State Key Laboratory of Vascular Homeostasis and Remodeling Peking University Beijing China; ^5^ Institute of Cardiovascular Diseases, The First Affiliated Hospital Dalian Medical University Dalian China; ^6^ Beijing Key Laboratory of Cardiovascular Receptors Research Beijing China; ^7^ Haihe Laboratory of Cell Ecosystem Beijing China; ^8^ MOE Frontier Science Center for Brain Research and Brain‐Machine Integration Zhejiang University School of Medicine Hangzhou China

## 1 A TREND OF DRUG DEVELOPMENT FOR THE APELIN RECEPTOR

**FIGURE 1:**
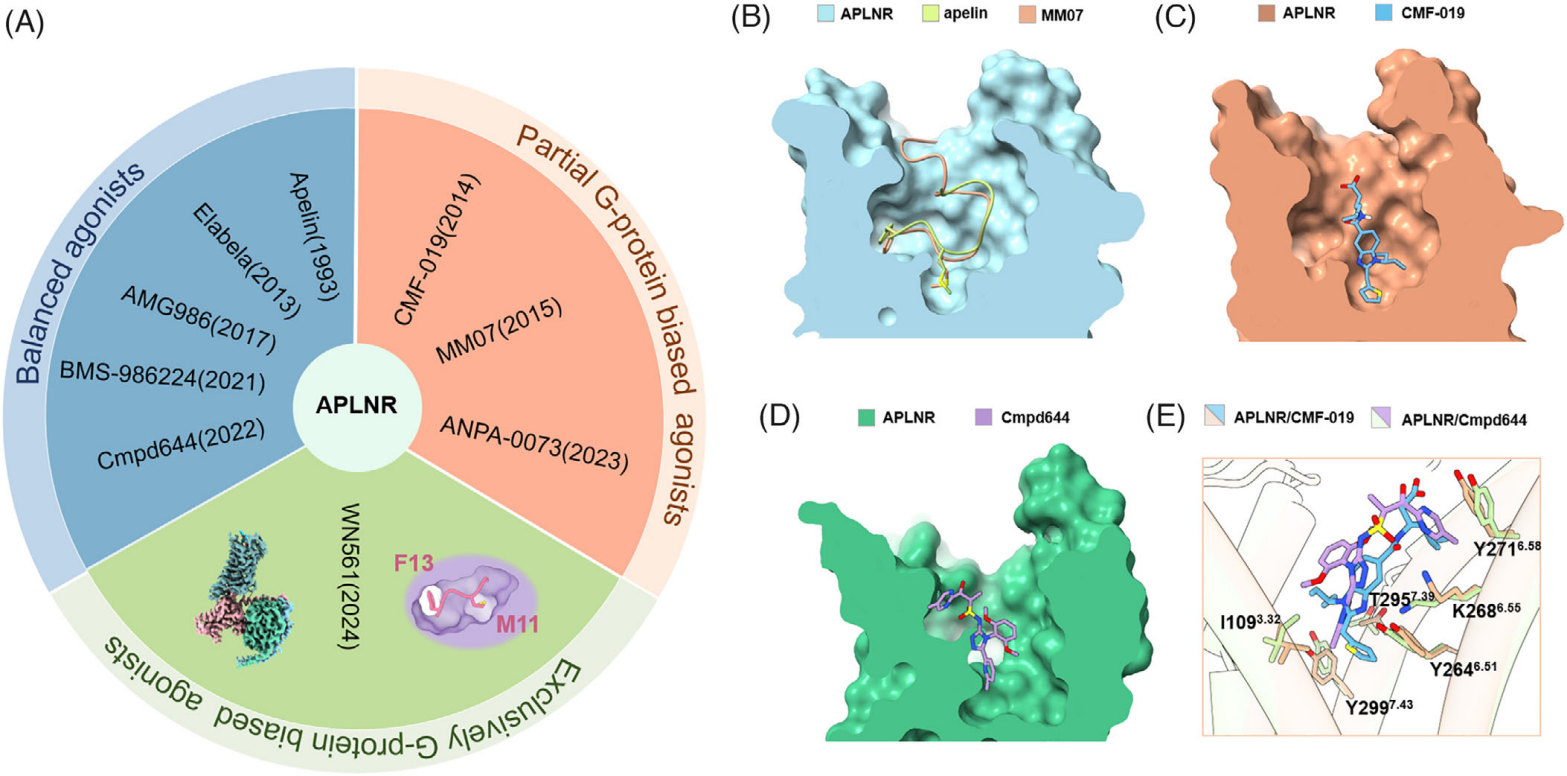
The structure resolution of apelin receptor (APLNR) promotes the development of exclusively G‐protein‐biased drugs. (A) APLNR agonists with divergent signalling profiles. Cross‐section of different agonists‐binding pockets in APLNR, such as (B) apelin and MM07, (C) CMF‐019 and (D) Cmpd644. (E) Comparisons of the detailed interactions of small‐molecule agonists CMF‐019 and Cmpd644 with APLNR, and residues that are key to the bias in APLNR are shown.

The apelin receptor (APLNR) plays many positive roles in the human body, especially in the cardiovascular system. Including facilitation of cardiac contractile force, enhancement of left ventricular stroke volume, vasodilation, promotion of diuresis, reduction of systemic blood pressure, anti‐hypertrophy and inhibit myocardial fibrosis,^1^, ^2^, ^3^, ^4^ making it a promising cardiovascular disease therapeutic target. As depicted in Figure 1A, the evolution from balanced agonists to exclusively G‐protein‐biased agonists has been fraught with challenges, with no approved drugs reaching the market to date. The challenge has been to mitigate the adverse effects of cardiac hypertrophy, which is mediated through β‐arrestin signalling downstream of the receptor, thereby limiting the pharmacological utility of these agonists. In the past decade, renowned universities and pharmaceutical companies have developed a series of small‐molecule agonists. Examples of such compounds include AMG986 (NCT03276728), BMS‐986224 (NCT03281122) and CLR325 (NCT02696967), all of which have been tested in vivo or clinical trials but have not yet shown clear therapeutic advantages for heart failure. The University of Cambridge has made strides in developing partial G‐protein‐biased APLNR agonists, MM07 and CMF‐019, which have shown promise in preclinical studies in rats and human models, albeit with reduced G‐protein activity compared to apelin.^5^, ^6^, ^7^, ^8^ However, the clinical development of these candidates has been met with setbacks, with the trial of MM07 terminated in phase I and no clinical studies initiated for CMF‐019. Safety concerns remain a pivotal issue for the advancement of APLNR‐targeted therapeutics into clinical trials. Therefore, does the development of absolute biased agonists possess the potential to provide superior therapeutic benefits? Furthermore, what strategies can be employed to successfully develop such biased agonists?

To solve this issue, Wang et al. have elucidated the key determinants of biased signalling in APLNR and have guided the design of exclusively G‐protein‐biased APLNR agonist WN561. As expected, WN561 shows superior therapeutic effects against cardiac hypertrophy and reduced adverse effects compared with the established APLNR agonists.^9^ This advancement propels the clinical application of apelin receptor agonists closer to realization.

## 2 STRUCTURAL GUIDANCE FOR EXCLUSIVELY G‐PROTEIN BIASED APLNR AGONISTS RATIONAL DESIGN

The elucidation of the APLNR‐biased signalling mechanism by Wang et al. not only guided the generation of absolute G‐protein‐biased peptide agonists but also reported the complex structure of the partial G‐protein‐biased small molecule CMF‐019 with the APLNR‐Gi protein complex, which is equally important for the development of absolute G‐protein‐biased small molecule agonists. When compared to the cryo‐electron microscopy structure of the APLNR‐Gi complex bound to the balanced small molecule agonist cmpd644, as reported by Yue et al. (Figure 1D),^9^, ^10^ indicates that CMF‐019 is structurally closer to TM6/7, consistent with the observations previously observed in the complex structure of peptide ligands. Moreover, the specific residues in the receptor that modulate biased signalling of small molecules are mostly consistent with the peptide agonists interacted with which they determined “twin hotspots” previously (Figure 1E).^9^ Therefore, modulating the interactions between small molecule groups and these amino acids can facilitate the development of modulators with absolute G‐protein bias. Given that WN561 demonstrates superior therapeutic advantages and reduced side effects in vivo, small molecule agonists with absolute G‐protein bias exhibit significant potential. Furthermore, these findings underscore the importance of structure‐based drug design in the refinement of biased agonists, highlighting the potential for targeted interventions in signalling pathways. The identification of these key residues and their role in mediating signalling bias provides a framework for the systematic optimization of future APLNR modulators. This strategic approach is likely to yield novel therapeutic agents that can selectively engage desired signalling pathways, thereby enhancing treatment efficacy and reducing the likelihood of adverse effects.

## 3 APLNR ACTS AS A MODEL FOR PRECISION DRUG DESIGN IN G PROTEIN‐COUPLED RECEPTOR‐TARGETED THERAPEUTICS

**FIGURE 2:**
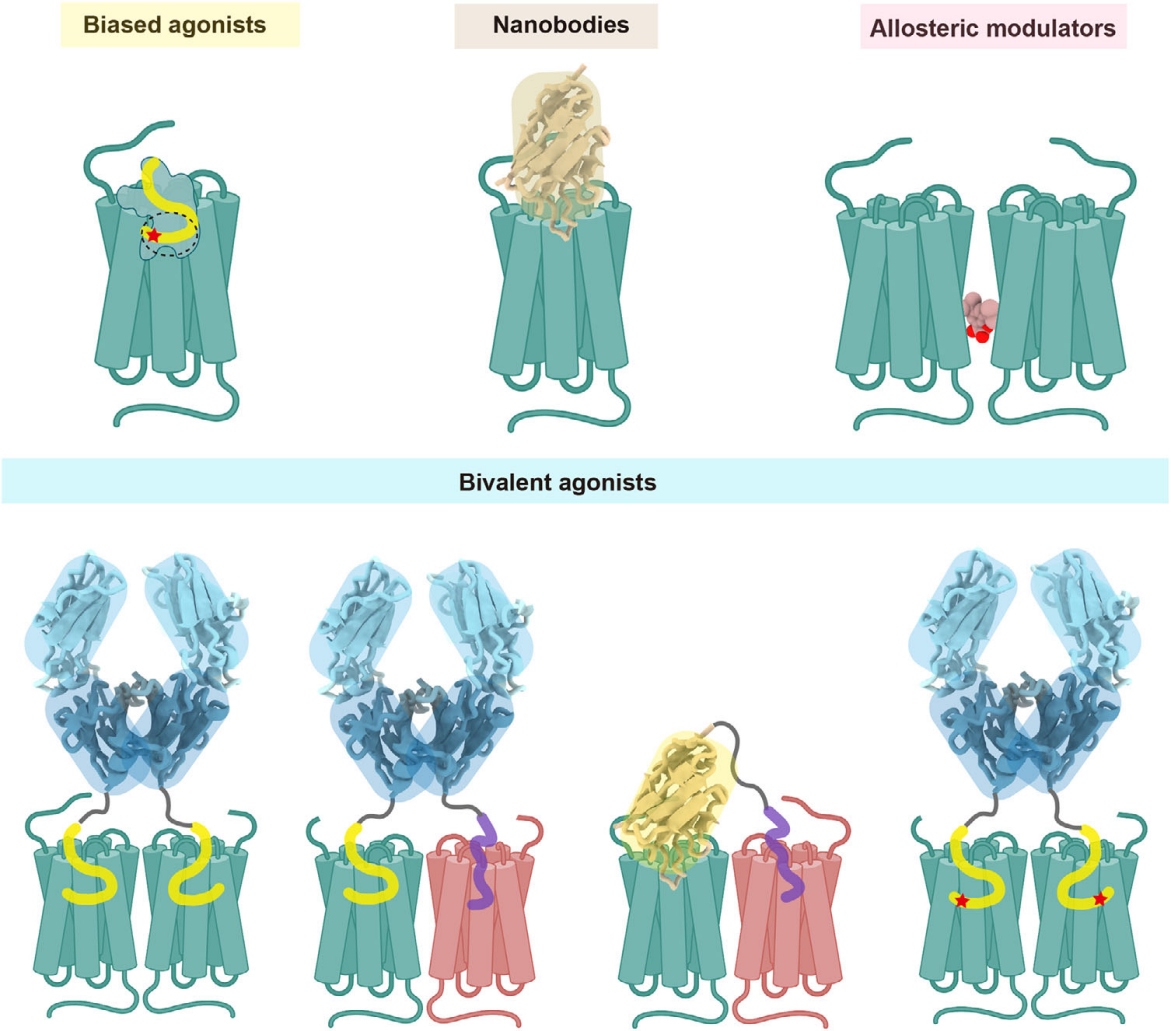
Structural blueprint for the design of diverse apelin receptor (APLNR) modulators. Red stars represent biased agonists.

Advances in G protein‐coupled receptor (GPCR) research have significantly enhanced our comprehension of biological processes, and the refinement of structural information and molecular mechanism of drug targets have brought precision drug design to fruition. APLNR serves as a paradigmatic research model, offering insights into the drug development of other receptors. Firstly, the successful development of its absolute signal‐selective agonists has catalyzed the exploration of biased drug signalling. Additionally, APLNR is the first receptor of Class A GPCR for which nanobodies have been developed,^11^ providing a theoretical basis for the development of nanobody modulators and application of nanobody‐conjugated bivalent agonists targeting different oligomeric forms (Figure 2). Furthermore, APLNR exhibits a variety of oligomeric forms in vivo, which perform diverse physiological functions. Notably, it is currently the only Class A GPCR for which the structure of an active APLNR complexed with heterotrimeric G‐protein has been elucidated.^10^ These structural insights offer a range of innovative strategies for future drug design targeting APLNR, such as signal‐biased agonists in the forms of peptides, small molecules, and nanobodies; allosteric modulators that regulate dimer formation; as well as bivalent agonists of homodimers and heterodimers that simultaneously modulating dual receptors (Figure 2). It provides a novel paradigm for the advancement of Class A GPCR‐targeted pharmaceuticals. In summary, Structure‐guided precision drug design will fill the technological gaps in precision medicine and provide novel strategies for the development of GPCR‐targeted drugs.

## AUTHOR CONTRIBUTIONS

Yan Zhang (ZJU), Weiwei Wang, Suyu Ji and Ping Xu conceptualized and wrote the commentary. Yan Zhang (PKU), Yan Zhang (ZJU) and Weiwei Wang provided the funding.

## CONFLICT OF INTEREST STATEMENT

The authors declare no conflict of interest.

## ETHICS STATEMENT

Not Applicable.
